# Supplementary figures and images for: A neuroendocrine pathway modulating osmotic stress in Drosophila
Source: PLoS Genet. 2021 Mar 8;17(3):e1009425. doi: 10.1371/journal.pgen.1009425 (PMC7971876; doi:10.1371/journal.pgen.1009425)

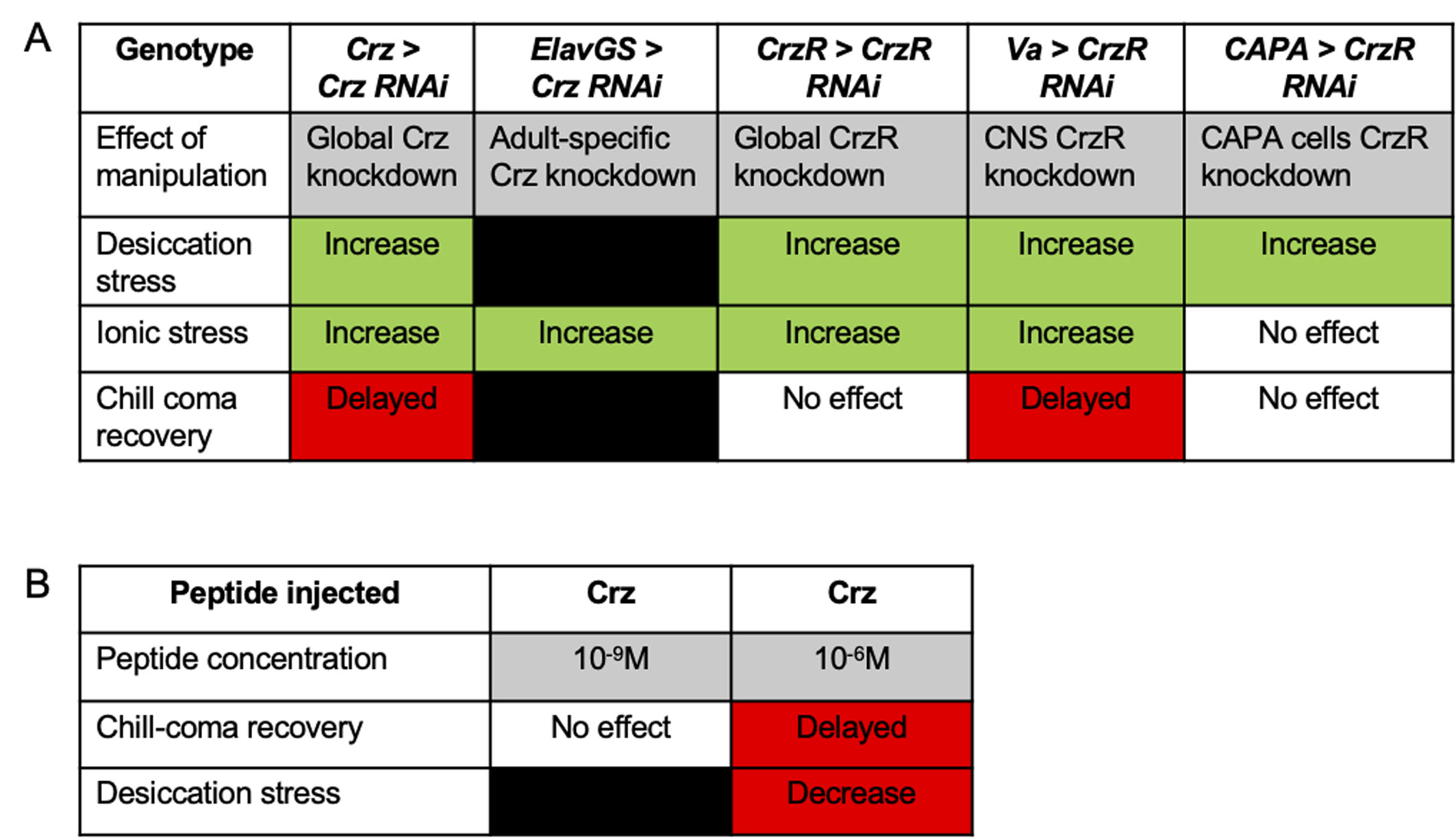

Supplement: S1 Table — (JPG) [file pgen.1009425.s002.jpg]

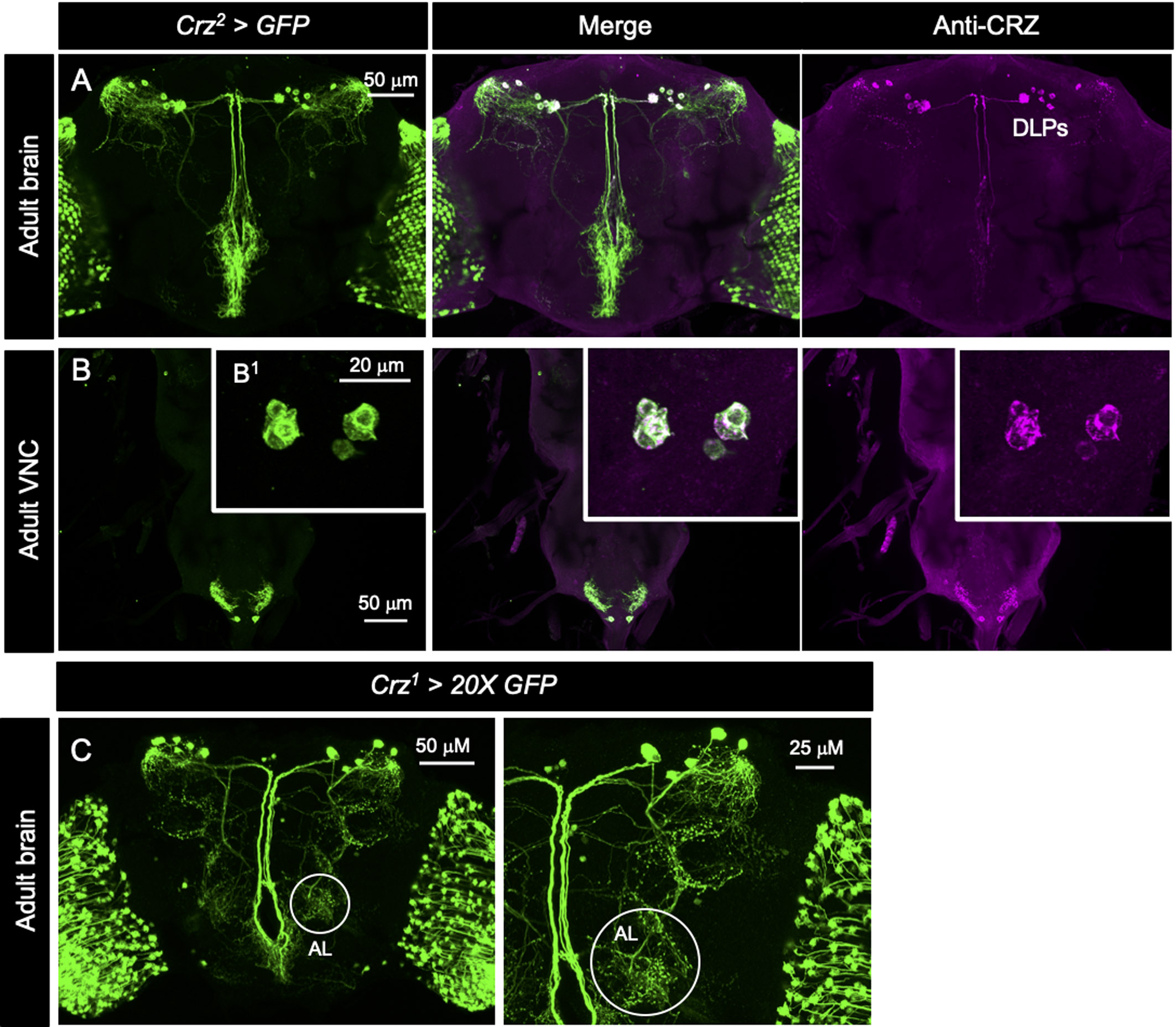

Supplement: S1 Fig — Crz2-GAL4 driven GFP and Crz-immunoreactivity is present in (A) dorsal lateral peptidergic neurons (DLPs) in the brain and (B) two to (B1) three pairs of male-specific neurons in the abdominal ganglia. (C) Crz1>20X GFP expression in the brain. Note that the Crz neuron arborizations can be seen in the antennal lobe (position marked with AL). (JPG) [file pgen.1009425.s003.jpg]

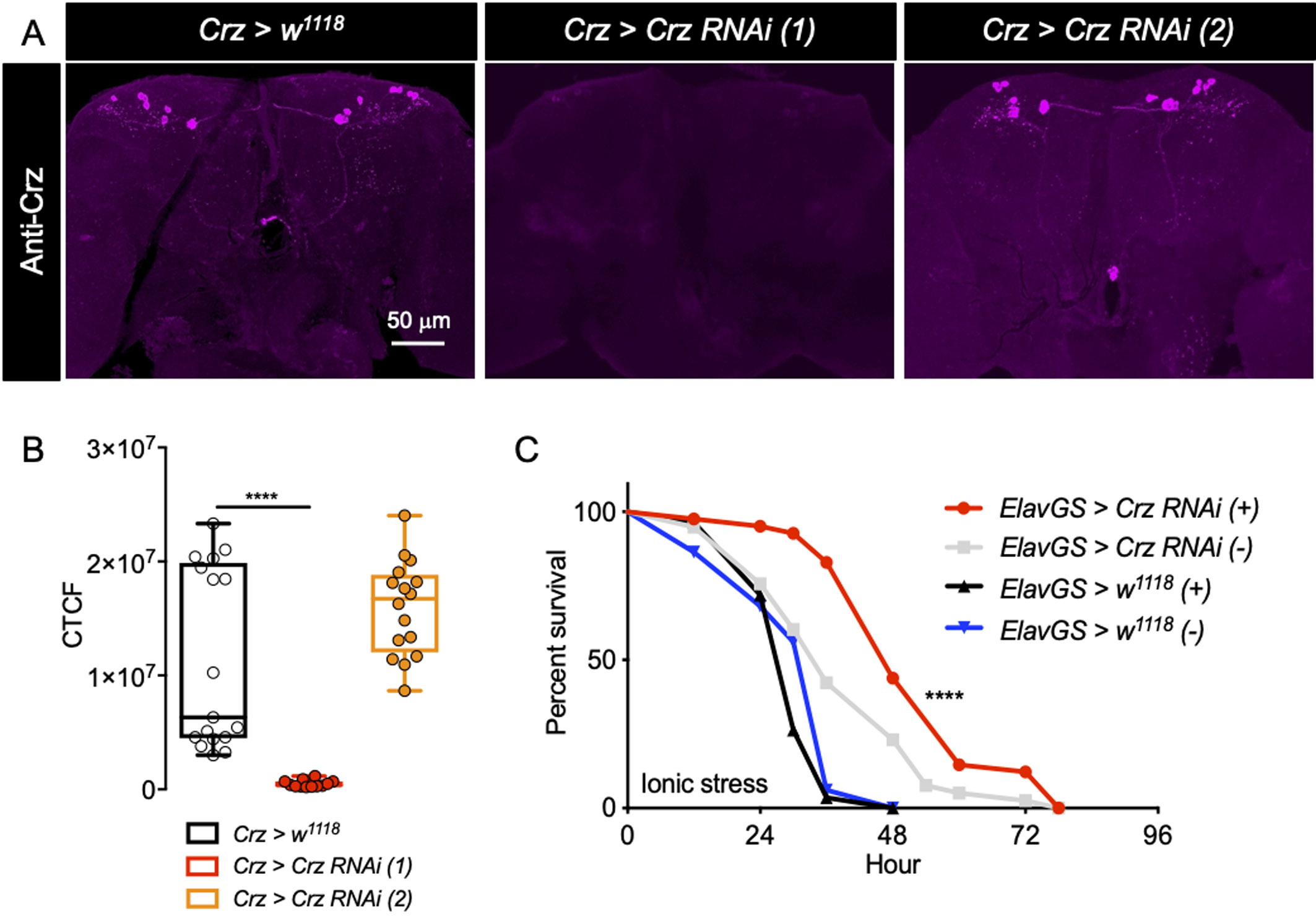

Supplement: S2 Fig — (A-B) Crz1-GAL4 driven Crz-RNAi (1) but not Crz-RNAi (2) causes a significant decrease in anti-Crz staining (corrected total cell fluorescence, CTCF) in the brains of adult Drosophila (**** p < 0.0001 as assessed by One-way ANOVA). (C) Adult-specific pan-neuronal knockdown of Crz results in increased survival under ionic stress. “+” indicates RU486 fed flies and “-”indicates control flies. Data are presented as survival curves (**** p < 0.0001, as assessed by Log-rank (Mantel-Cox) test). (JPG) [file pgen.1009425.s004.jpg]

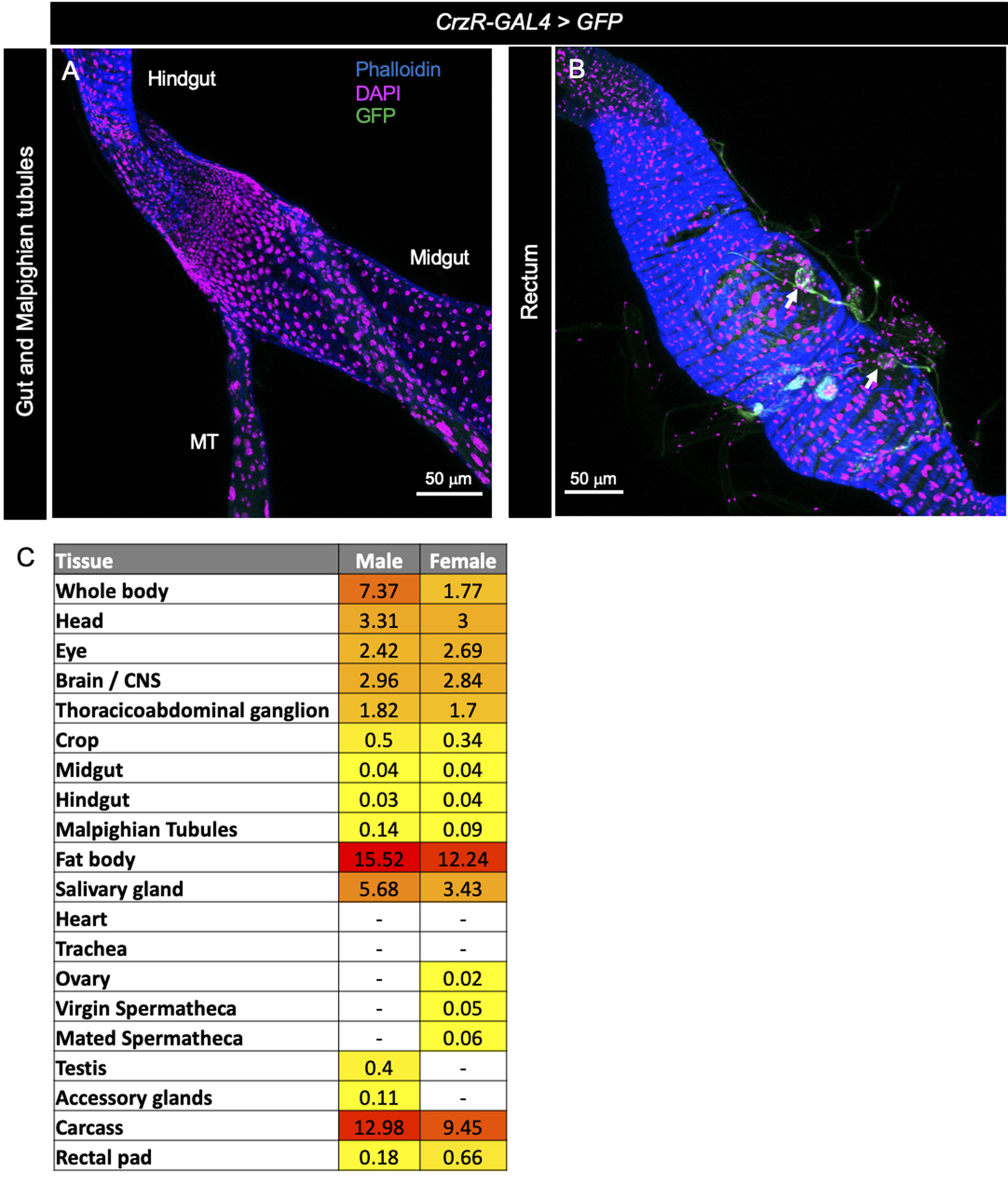

Supplement: S3 Fig — (A) CrzR-GAL4 driven GFP is not expressed in the midgut, hindgut and Malpighian tubules (MTs). (B) CrzR is weakly expressed in neurons innervating the rectum. White arrows indicate GFP staining in the region, presumably the water-resorbing rectal pads, which does not stain for phalloidin. (C) In adults, CrzR is not expressed in tissues (hindgut, MTs and rectal pad) associated with ionic and osmotic homeostasis (Data assembled from FlyAtlas 2). (JPG) [file pgen.1009425.s005.jpg]

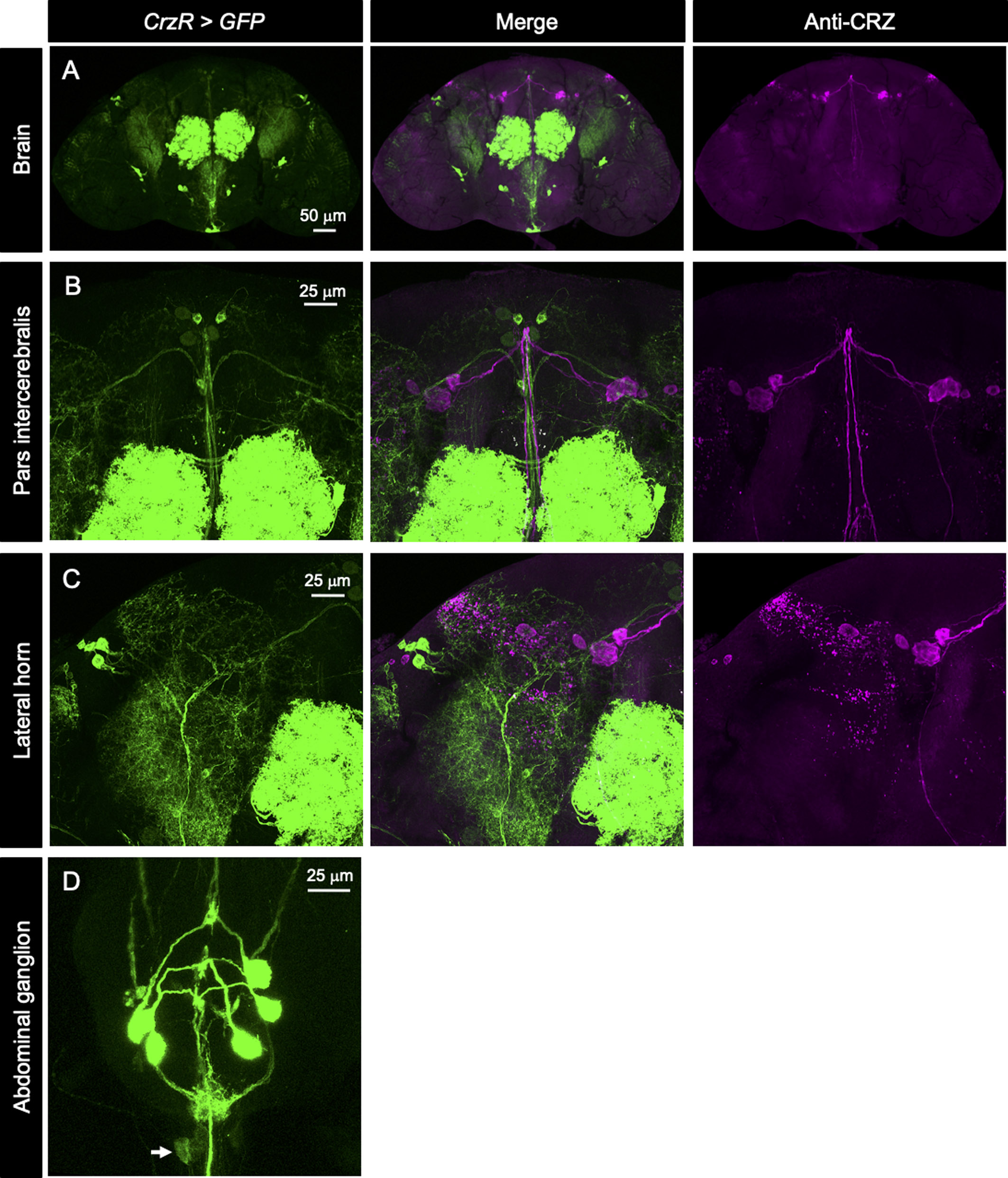

Supplement: S4 Fig — (A, B) Crz-producing dorsal lateral peptidergic neurons (DLPs) send projections to the pars intercerebralis near a set of CrzR>GFP expressing median neurosecretory cells. (C) Crz interneuron projections partly overlap CrzR>GFP expressing processes in the lateral horn. In A-C, note high CrzR expression (based on GFP intensity) in the local interneurons innervating the antennal lobe. (D) CrzR-GAL4 drives strong GFP expression in three pairs of neurons in abdominal ganglion. Weak GFP expression is also observed in two pairs of neurons (indicated by a white arrow). (JPG) [file pgen.1009425.s006.jpg]

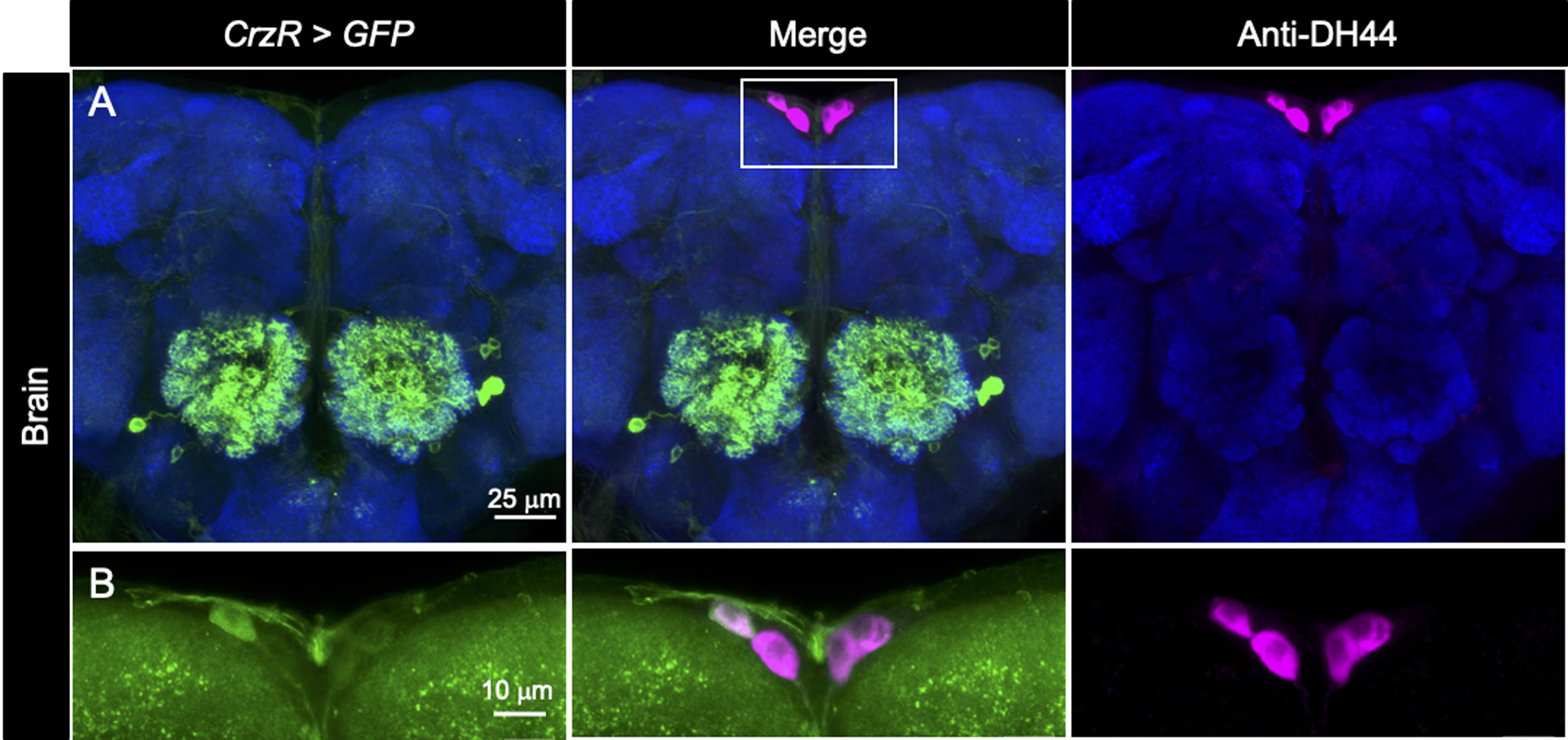

Supplement: S5 Fig — (A) CrzR-GAL4 drives GFP expression in the median neurosecretory cells (MNCs) expressing diuretic hormone 44 (DH44) neuropeptide. (B) Higher magnification image of the DH44 MNCs (white box in A). Note that the GFP expression in DH44 neurons is weak and variable. (JPG) [file pgen.1009425.s007.jpg]

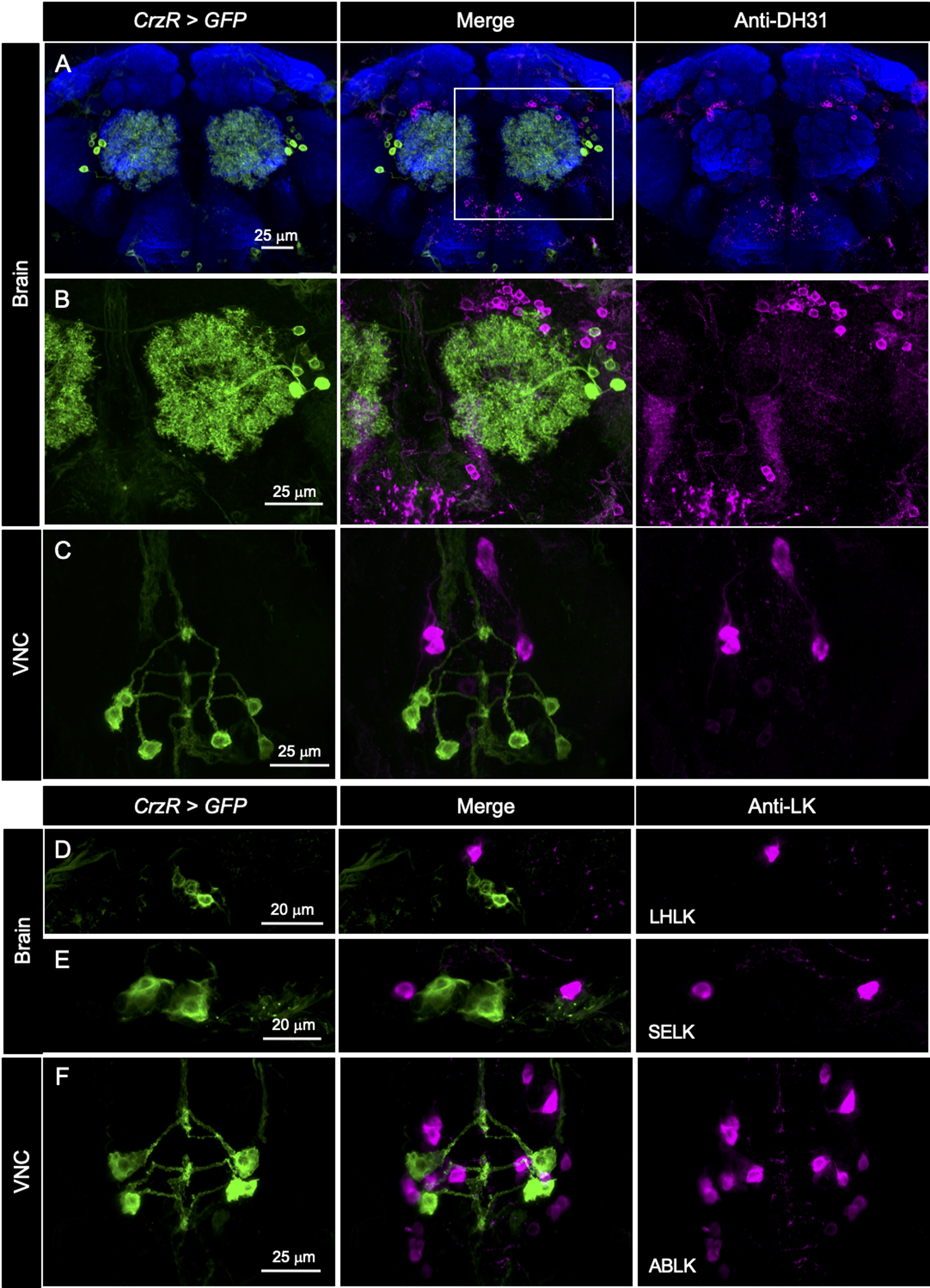

Supplement: S6 Fig — CrzR-GAL4 driven GFP is not expressed in diuretic hormone 31 (DH31) neurons of (A, B) the brain and (C) the ventral nerve cord. B shows a higher magnification image of the DH31 neurons surrounding the antennal lobe MNCs (white box in A). CrzR-GAL4 driven GFP is not expressed in leucokinin (Lk) neurons of (D) the lateral horn (LHLKs), (E) subesophageal zone (SELKs) and (F) the abdominal ganglia (ABLKs). (JPG) [file pgen.1009425.s008.jpg]

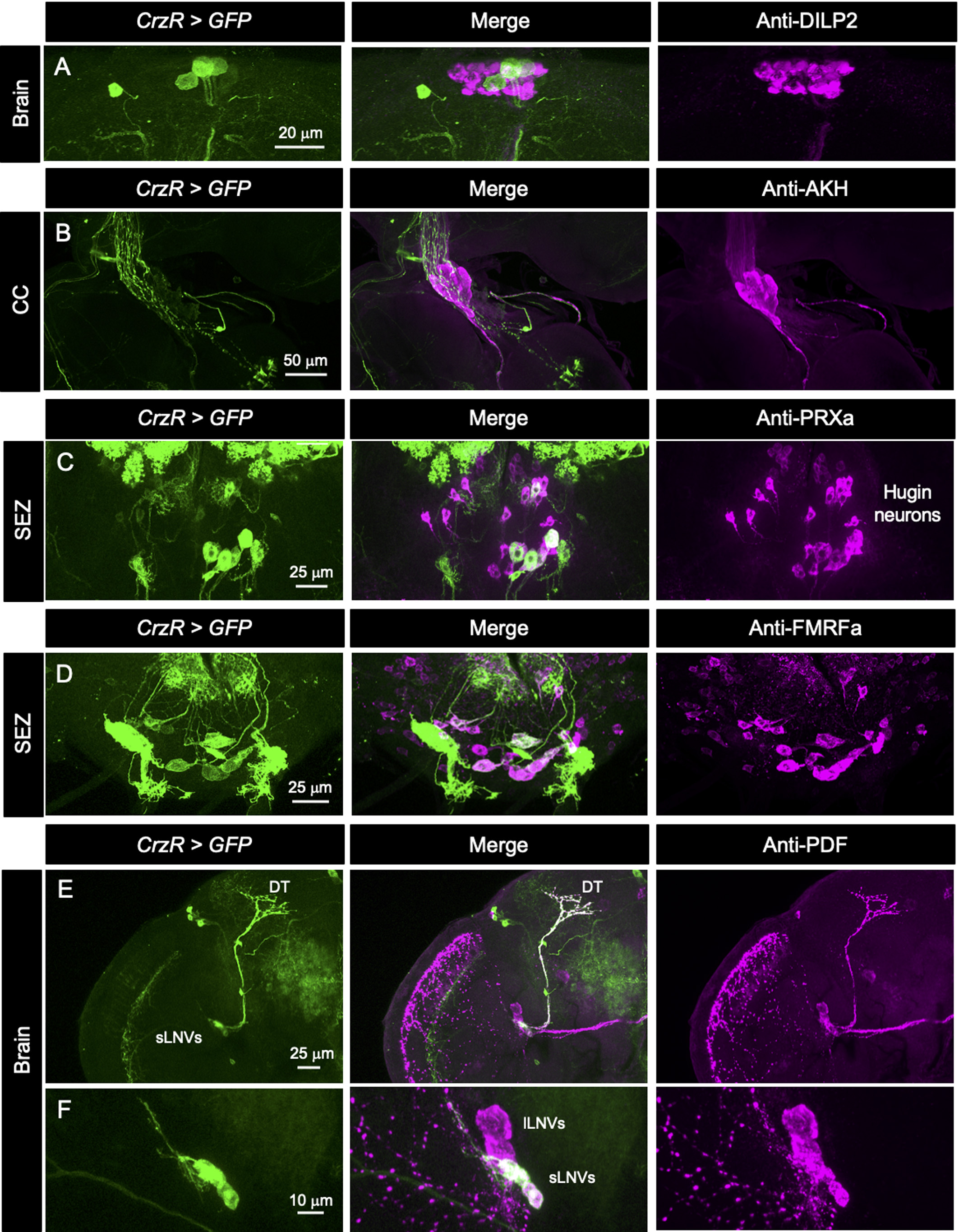

Supplement: S7 Fig — CrzR is not expressed in (A) brain neurosecretory cells expressing Drosophila insulin-like peptide 2 (DILP2) and (B) adipokinetic hormone (AKH)-producing cells of the corpora cardiaca (CC). CrzR-GAL4 drives GFP expression in (C) Hugin neurons and (D) neurons expressing FMRFamide-related peptide in the SEZ. Note that the exact identity of the neuropeptide present in the neurons labelled with FMRFamide antibody is unknown as it cross-reacts with multiple neuropeptides (E, F) CrzR-GAL4 drives GFP expression in sLNv clock neurons labeled with anti-pigment dispersing factor (PDF) antibody. CrzR>GFP expression is present in the characteristic dorsal-projecting (DT) axons of the sLNvs. (JPG) [file pgen.1009425.s009.jpg]

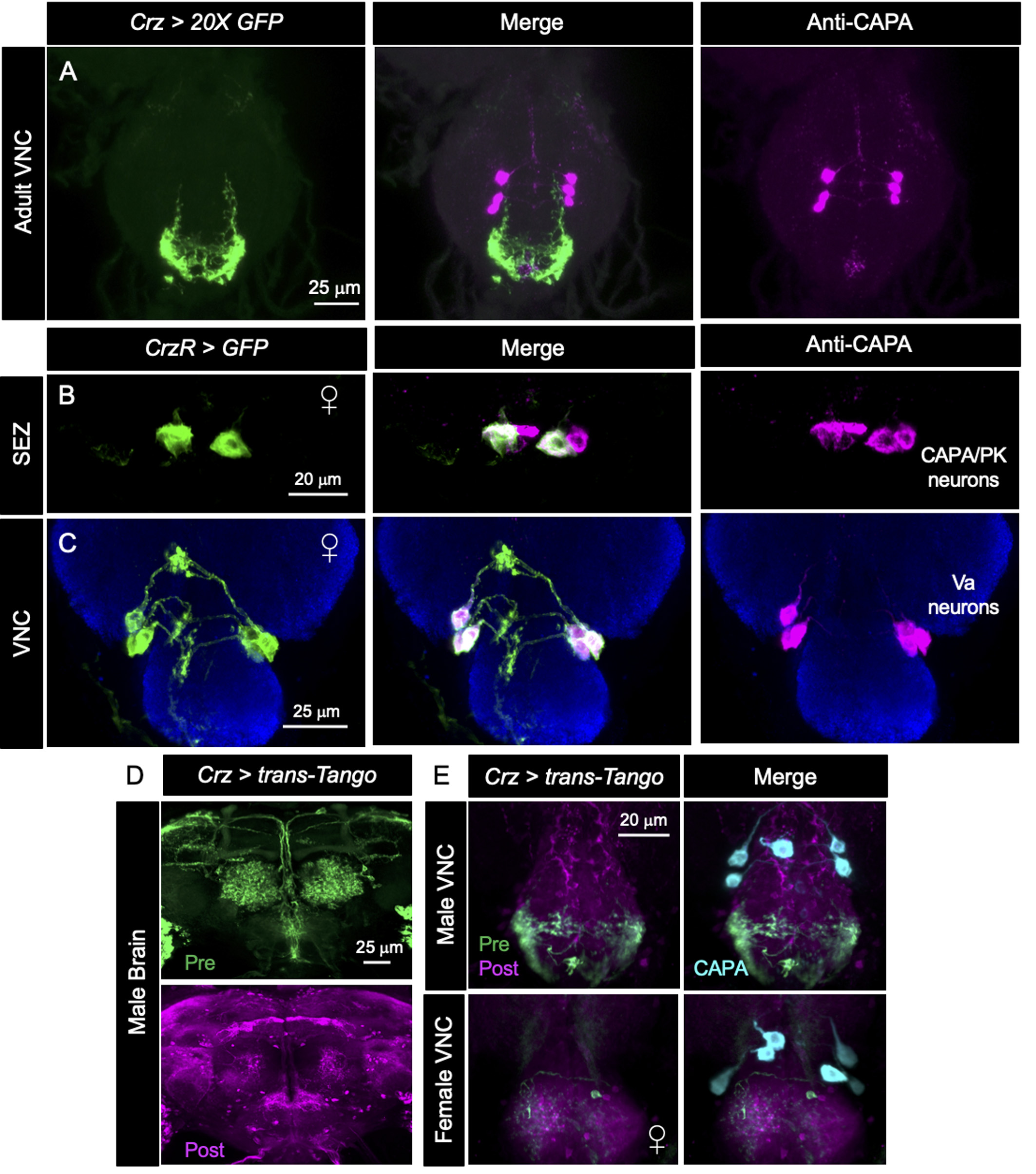

Supplement: S8 Fig — (A) Crz interneurons in the abdominal ganglion send axon projections in close proximity to Va neurons. CrzR-GAL4 drives GFP expression in CAPA/pyrokinin (CAPA/PK) producing neurons (labeled with anti-CAPA antibody) in (B) the subesophageal zone (SEZ) and (C) ventral nerve cord (VNC) of adult females. The three pairs of neurons in the VNC are referred to as Va neurons. (D) Crz-GAL4 driven trans-Tango generates presynaptic signal (labeled with anti-GFP antibody) in the DLPs and a postsynaptic signal (labeled with anti-HA antibody) in various regions of the brain. (E) Crz > trans-Tango postsynaptic signal is absent in Va neurons of both males and females. (JPG) [file pgen.1009425.s010.jpg]

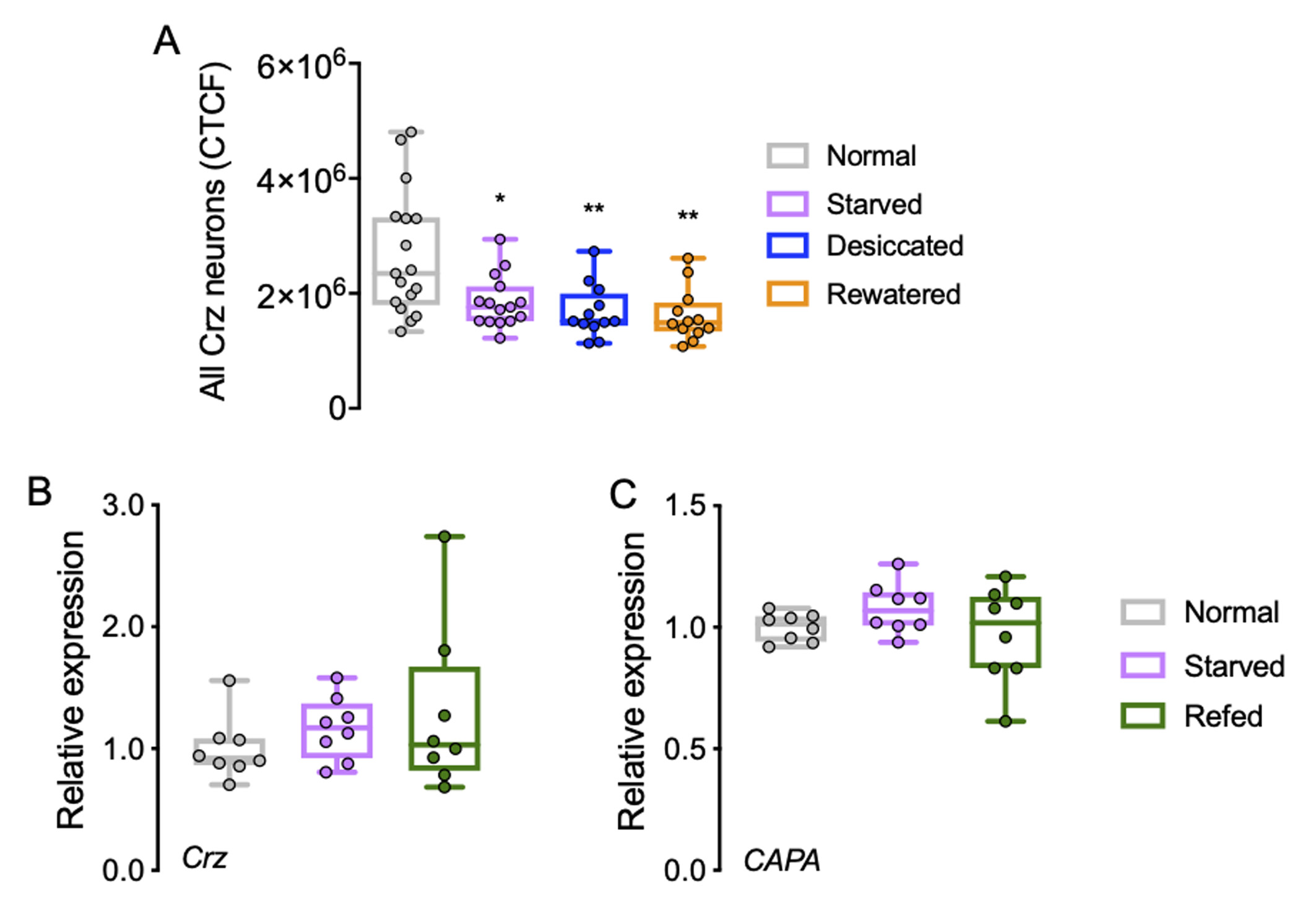

Supplement: S9 Fig — (A) Adult flies were either kept under normal conditions, starved, desiccated or rewatered (desiccated and then incubated on 1% aqueous agar) and Crz peptide levels monitored using immunohistochemistry. Crz peptide levels in all Crz neurons (see Fig 4A for representative images) are lower in starved, desiccated and rewatered flies compared to flies raised under normal conditions (* p < 0.05, ** p < 0.01 as assessed by One-way ANOVA). Starvation and refeeding do not impact (B) Crz and (C) CAPA transcript levels. (JPG) [file pgen.1009425.s011.jpg]

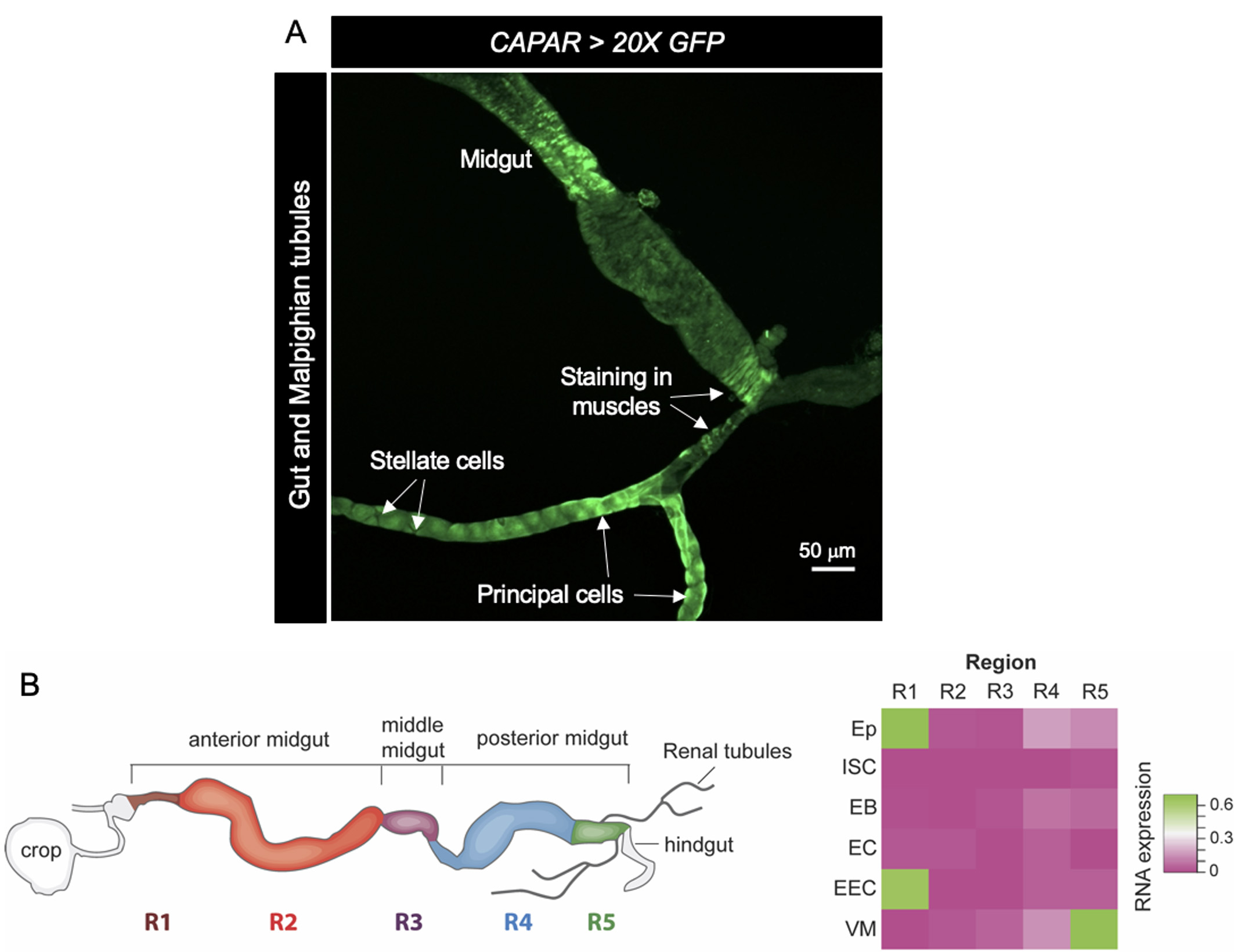

Supplement: S10 Fig — CAPAR-GAL4 drives 20X GFP (pJFRC81-10xUAS-Syn21-myr::GFP-p10) expression in the adult (A) principal cells of the Malpighian tubules as well as in gut muscles. Note the lack of GFP staining in star-shaped stellate cells. (B) A schematic of the adult gut and heat map showing expression of CAPAR in different regions of the gut (R1 to R5) and its various cell types (VM, visceral muscle; EEC, enteroendocrine cell; EC, enterocyte; EB, enteroblast; ISC, intestinal stem cell; Ep, epithelium. Data was mined using Flygut-seq. The CAPAR-GAL4 expression pattern is in agreement with the transcriptomic data. (JPG) [file pgen.1009425.s012.jpg]

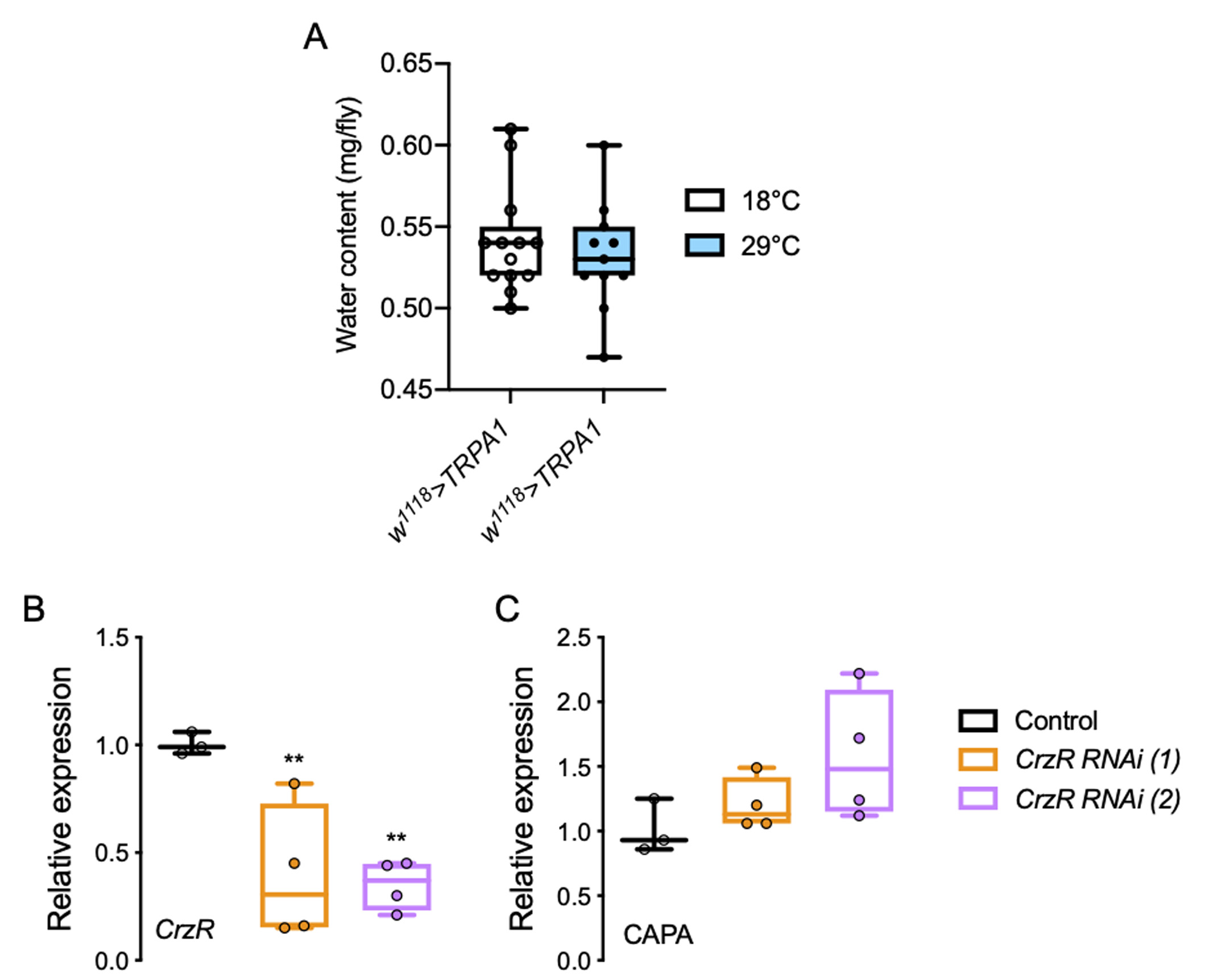

Supplement: S11 Fig — (A) There is no difference in the water content of w1118 > TRPA1 flies incubated at 18°C and 29°C. (B) Actin-GAL4 driven CrzR-RNAi (two independent RNAi lines) results in efficient knockdown of CrzR transcript in whole adult flies compared to control flies (Actin>w1118) as tested by qPCR. (** p < 0.01 as assessed by One-way ANOVA). (C) Actin-GAL4 driven CrzR-RNAi has no impact on Capa transcript levels compared to control flies (Actin>w1118). (JPG) [file pgen.1009425.s013.jpg]

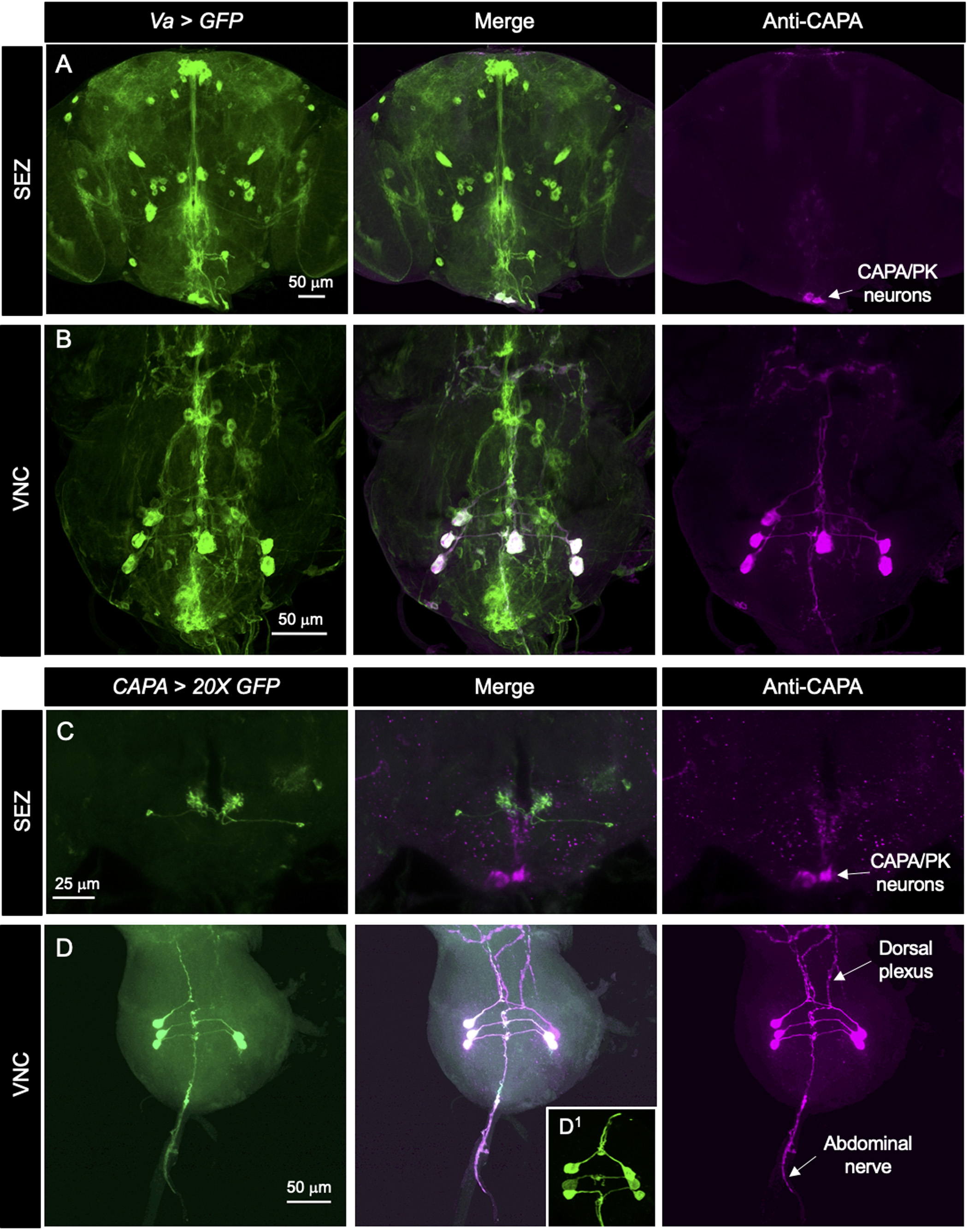

Supplement: S12 Fig — (A) Va-GAL4 drives GFP expression in several neurons in the central nervous system, including a pair of CAPA/pyrokinin producing neurons in the SEZ and (B) Va neurons in the VNC (labeled with anti-CAPA antibody). (C) CAPA-GAL4 does not drive GFP expression in the CAPA/pyrokinin neurons in the SEZ but it does so in Va neurons in the VNC (D). Only 5 neurons are visible in this preparation; however, there are usually 6 neurons in most preparations (D1). Note that the posterior-most pair of Va neurons send axonal projections into the abdominal nerve (indicated by the white arrow) where they terminate to form neurohemal release sites. The anterior two pairs send axons to a neurohemal plexus in the dorsal neural sheath. (JPG) [file pgen.1009425.s014.jpg]

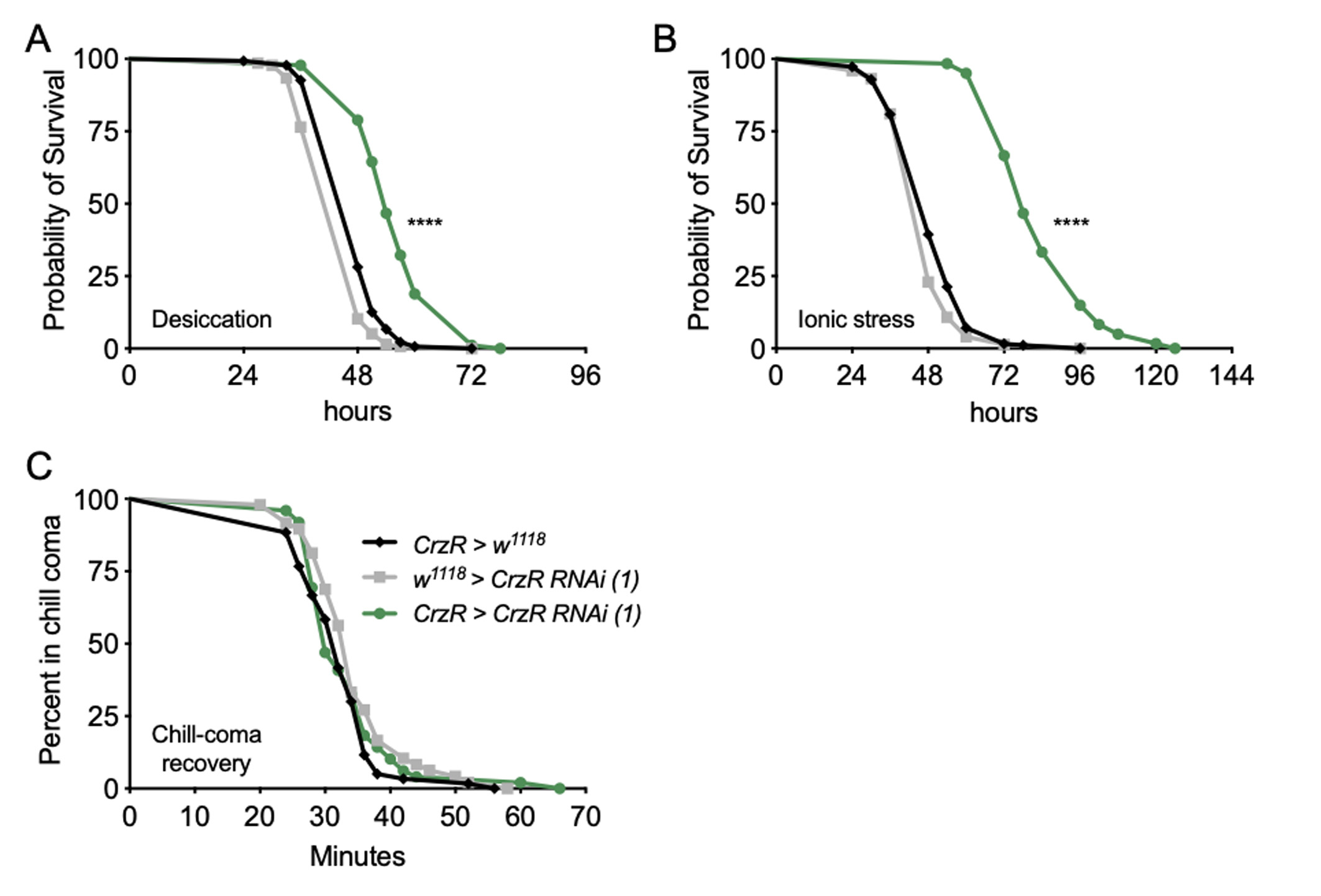

Supplement: S13 Fig — CrzR-GAL4 driven CrzR-RNAi(1) results in (A) increased survival under desiccation and (B) ionic stress but has no impact on (C) chill-coma recovery. Data are presented as survival curves (**** p < 0.0001, as assessed by Log-rank (Mantel-Cox) test). (JPG) [file pgen.1009425.s015.jpg]

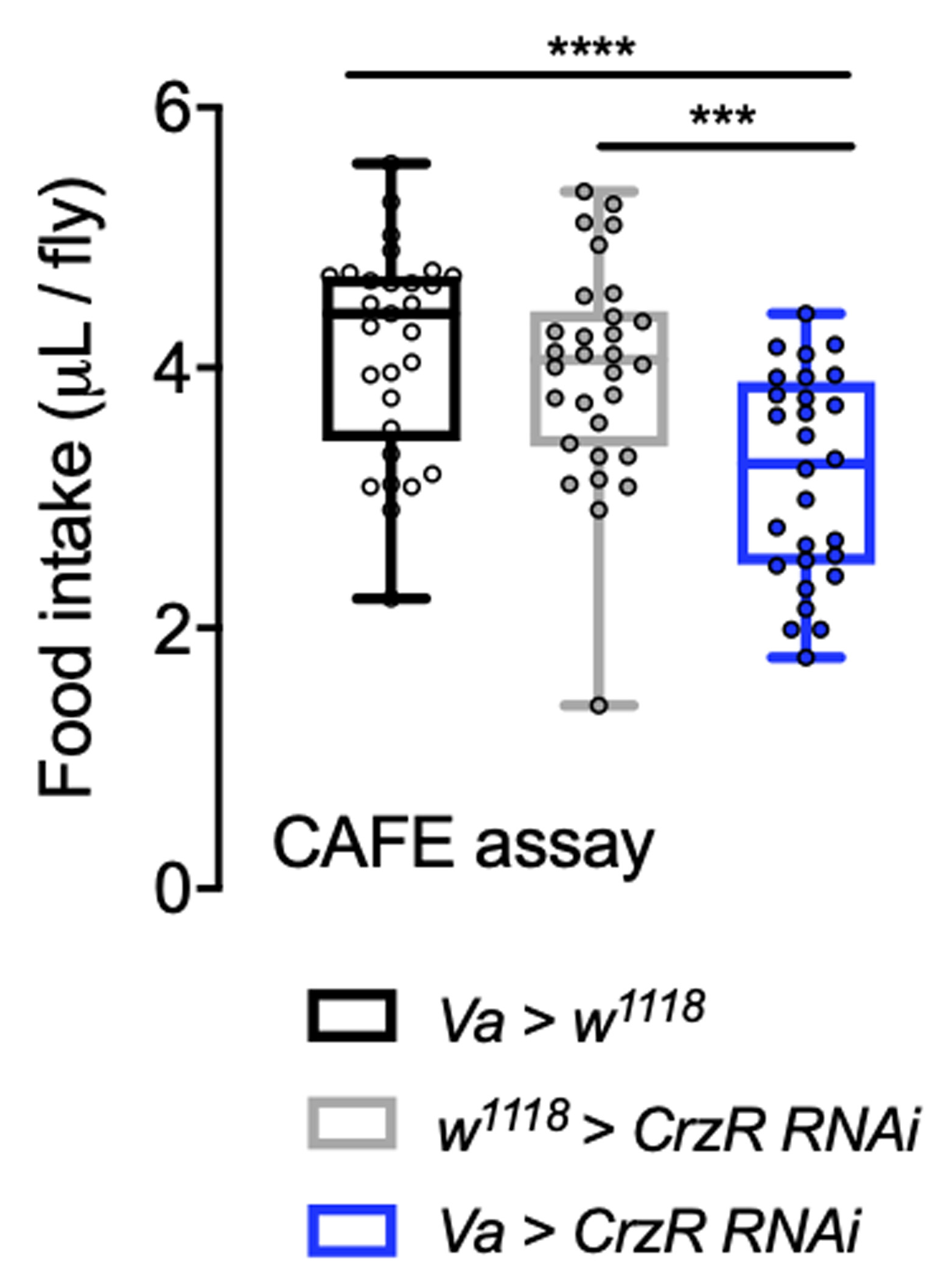

Supplement: S14 Fig — CrzR knockdown in Va neurons results in reduced cumulative food intake measured with CAFE assay over 4 days (*** p < 0.001, **** p < 0.0001 as assessed by One-way ANOVA). (JPG) [file pgen.1009425.s016.jpg]
